# Introduction to the *RSC Advances* Emerging Investigators series 2021

**DOI:** 10.1039/d2ra90072f

**Published:** 2022-08-12

**Authors:** James Batteas

**Affiliations:** Department of Chemistry, Texas A&M University College Station TX 77843-3255 USA

## Abstract

*RSC Advances* is proud to present the 2021 Emerging Investigators series. Guest Edited by Professor James Batteas (Texas A&M University), this series showcases some of the very best work from chemists in the early stages of their independent careers.
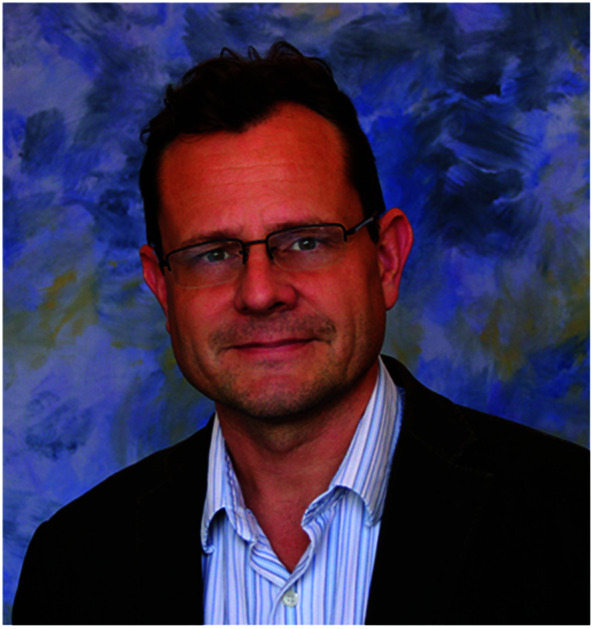

We are so pleased to introduce this first issue of our *RSC Advances* Emerging Investigators series! Launched in 2021, the series seeks to highlight up and coming researchers. Selection for the Emerging Investigators series comes in part from the recommendations of our Editorial Board as well as our Associate Editors. Authors can also self-nominate for participation and review by our Associate Editors for the journal. In keeping with the theme of *RSC Advances* as a cross-cutting chemistry journal, in this inaugural issue we have 23 papers spanning the breadth of chemistry on topics ranging from the development and application of analytical tools and devices for chemical analysis, to the design and synthesis of bioactive materials for disease treatments, to catalysis and synthesis of new materials.

We introduce the 2021 series with a Review article by Dusselier *et al.* (https://doi.org/10.1039/D1RA02887A) which discusses the ambiguity in our current understanding of the mechanisms behind interzeolite conversion as a technique for the synthesis of zeolite frameworks. This article highlights how heteroatoms such as aluminium have important roles in this process.

We then launch into a series of analytical contributions to introduce the primary research papers in this collection. Paixão *et al.* (https://doi.org/10.1039/D0RA08874A) explore the development of electrochemical paper-based analytical devices for low-cost electrochemical sensing, in which even pencil-drawn electrodes can be used for device fabrication. Lewis *et al.* (https://doi.org/10.1039/D1RA04470B) show how 2D tin sulfide nanosheets can be assembled using simple Langmuir–Blodgett techniques to create novel, solution processed photodetectors. Next, Lucena *et al.* (https://doi.org/10.1039/D1RA02721B) discuss how chemically functionalized hypodermic needles can be used for selective extraction of tricyclic antidepressant (TCA) drugs from oral fluid as a simple and convenient approach for in-needle microextractions, allowing the samples to be further rapidly analyzed by mass spectrometry. Rapid screening of chemical compounds is of course an essential component of understanding and identifying materials for protein assays. To this end, Trader *et al.* (https://doi.org/10.1039/D0RA10976B) developed one-bead-one-compound (OBOC) libraries in which a target oncoprotein, gankyrin, is labeled with a NIR range fluorophore, and screened against a 343-member peptoid library to afford a method to quickly identify quality binders to a target protein of interest. Tsai *et al.* (https://doi.org/10.1039/D1RA04875A) developed an acoustofluidic method for the detachment of cells adhered onto a microchannel surface, without exposing the cells to any enzymatic or non-enzymatic compounds. This approach demonstrates a rapid, easy-to-operate, and cost-effective means of detaching or probing the adhesion strength of different cell types in various lab-on-a-chip applications. Lastly, Gupta *et al.* (https://doi.org/10.1039/D1RA08610C) prepare hydrogel gratings with an analyte responsive dye. The patterned dye is its own dispersive element, and therefore can enable the gratings to provide spectroscopic information without the use of external spectrophotometers.

Synthesis of new materials is of course a cornerstone of chemistry, and in the work highlighted in this issue, we see studies from Rodrigues *et al.* (https://doi.org/10.1039/D0RA10859F) in which carbon dots (C-dots) could be synthesized from a variety of carbon sources, which when functionalized with phenylboronic acid (PBA) yielded a highly efficient glucose sensor *via* bioimaging, and also offer the potential for cancer treatment as they could be used to induce necrosis in tumor tissues in cancer-bearing mice. Continuing in the theme of cancer treatments, Pianowski *et al.* (https://doi.org/10.1039/D0RA08893E) demonstrated that nontoxic supramolecular low-MW hydrogels could be used to quickly and selectively release potent anticancer agents upon light irradiation. Similarly, Sarkar *et al.* (https://doi.org/10.1039/D1RA01660A) synthesized an amphiphilic polymer, poly(PEGMA-*co*-SEMA) (BCP), by controlled reversible addition fragmentation chain transfer (RAFT) polymerization. The core–shell supramolecular assembly polymeric nano-architecture formed could be used as an efficient delivery system for anticancer drugs like doxorubicin (DOX). While polymeric materials can act as drug delivery agents, they can also act as protective coatings, as Heredia *et al.* (https://doi.org/10.1039/D1RA03417K) show in their work in which antimicrobial coatings could be created using films of PEDOT–fullerene C_60_ polymeric dyads. These films enable surface coatings that are self-sterilizing, as pathogens can be inactivated using the photodynamic activation of reactive oxygen. Here the authors demonstrated photosensitized inactivation by the electropolymerized films on bacteria suspensions with a >99.9% reduction in *S. aureus* survival. Finally, Pal *et al.* (https://doi.org/10.1039/D1RA07425C) develop some hydrophobic polymers with low glass transition temperatures that can be used in photovoltaic devices, where systems with smart polymeric coatings can optimize durability and efficacy. The polymers that they develop display photo-regulated self-healing mechanisms.

Sustainable synthesis also continues to be a major theme in chemistry and in this issue, Egbedina *et al.* (https://doi.org/10.1039/D1RA01130H) use ZnO biochar (made from coconut husks, kaolinite and ZnCl_2_) to make materials for wastewater treatment to remove unused antibiotics. Cleanup of plastics from water is also a critical challenge facing us, and Tiwary *et al.* (https://doi.org/10.1039/D1RA03097C) show how a schwarzite based 3D-printed water filter can be used for the cleanup of nanoplastics. But sustainability goes beyond just remediation. Hahn *et al.* (https://doi.org/10.1039/D1RA03692K) show that the immobilization of polyketide synthase through crosslinking could yield recyclable catalysts for chiral synthesis. In addition to being environmentally more benign, cost savings through the design of new scalable catalysts are also impactful. Zhang *et al.* (https://doi.org/10.1039/D1RA04112F) show us how efficient cross-coupling of aryl halides can be accomplished with high selectivity and low cost using a new atomically dispersed copper catalyst (Cu–ZnO–ZrO_2_).

The design of materials also takes on key challenges in discovering critical structure/function relationships. For example, Luo *et al.* (https://doi.org/10.1039/D1RA03366B) examine how plastic crystals of neopentyl glycol (NPG) can exhibit high ionic conductivities, making them promising candidates for applications in fuel cells, batteries, and supercapacitors. In terms of controlling unique optical properties of materials, Leitao *et al.* (https://doi.org/10.1039/D1RA02961D) was able to synthesize stable naphthalene bridged disilanes, capable of forming stable excimer complexes in non-polar solvents. The materials provide opportunities to create novel light emitting devices and/or photocurrent generators for solar cells. Around the development of supramolecular luminescent materials in water, hydrophobic chromophores can present a number of issues that limit their performance in aqueous media. Research in this collection by Xiao *et al.* (https://doi.org/10.1039/D1RA06239E) describes the development of a water-phase artificial light-harvesting system that can work efficiently in water, opening up more possibilities for dynamic luminescent materials.

The development of artificial supramolecular systems that can mimic the power of enzymes is an important field of development for a number of applications. In the contribution by Hu *et al.* (https://doi.org/10.1039/D1RA07958A), two water-soluble pillar[5]arenes with peripheral rims that contain opposite charges are prepared, and investigation of their influence on the Kemp elimination reaction of 1,2-phenylisoxazole derivatives was studied, helping to advance this area of research. Insight into reaction mechanisms using computational studies is central to advancing chemistry. Wang *et al.* (https://doi.org/10.1039/D1RA04564D) use multi-scale simulations to expand our understanding of P450-catalyzed C(sp^3^)–H amination reactions, through an optimized understanding of the P450-mediated amination of the pyrrolidine derivative of lidocaine. Globisch *et al.* (https://doi.org/10.1039/D1RA05994G) carry out analysis of the sulfated metabolites in various biological samples, as sulfated metabolites have been identified as being an identifier of the co-metabolism between the microbiome and its host. Through this, they are able to identify the core sulfatome of 41 metabolites. This brings us to our final contribution to this issue from Beemelmanns *et al.* (https://doi.org/10.1039/D1RA00997D) which highlights how using data screening methods such as Global Natural Products Social Molecular Networking Analysis (GNPS) can reveal key bioactive molecular species, in this case focused on metabolic studies of *Pseudoxylaria* sp. X187.

We hope that you find this issue inspirational for both the breadth and high quality of chemistry described by these up-and-coming researchers, and we look forward to expanding this series in the years to come.

## Supplementary Material

